# A Practical Rule-of-Thumb to Adapt Contrast Media Dose in Photon-counting Detector CT: The 10-to-5 Rule

**DOI:** 10.1097/RLI.0000000000001253

**Published:** 2025-11-26

**Authors:** Cécile R.L.P.N. Jeukens, Bibi Martens, Janneke Vandewall, Sören Jasper, Guy Schrijnemaekers, Joachim E. Wildberger, Thomas G. Flohr

**Affiliations:** Department of Radiology and Nuclear medicine, Maastricht University Medical Centre+, Maastricht, The Netherlands (C.R.L.P.N.J., B.M., J.V., S.J., G.S., J.E.W., T.G.F.); Research Institute for Oncology and Reproduction (GROW), Maastricht University, Maastricht, The Netherlands (B.M.); Siemens Healthineers AG, Diagnostic Imaging, Computed Tomography Research & Development, Forchheim, Germany (S.J., T.G.F.); Cardiovascular Research Institute Maastricht (CARIM), Maastricht University, Maastricht, The Netherlands (J.E.W.)

**Keywords:** computed tomography, diagnostic imaging, abdomen PVP, CTA, contrast media, photon-counting CT, injection protocol

## Abstract

**Objectives:**

To develop a simple rule-of-thumb on how to reduce the contrast medium (CM) dose in photon-counting detector CT (PCD-CT) when lowering the energy of the reconstructed virtual mono-energetic images (VMI) while maintaining the contrast-to-noise ratio (CNR) for parenchymal CT and CTA.

**Materials and Methods:**

Spectral abdominal and chest CT phantoms were scanned using a portal venous phase (PVP) abdominal and a high-pitch CTA protocol, respectively, on a first-generation dual-source PCD-CT. The phantoms contained cylindrical rods with iodine in water equivalent material (0.5/1.0/2.0/5.0/10.0/15.0 mg I/mL) and ICRU muscle tissue. The phantoms were complemented with 2 fat equivalent rings to mimic different patient sizes. Iodine contrast, image noise, noise power spectra (NPS), and iodine CNR were investigated in VMIs with different energies (40 to 60 keV in steps of 5 keV). This was done for different iodine concentrations, phantom sizes, x-ray tube voltages (120 kV and 140 kV) and radiation doses. In addition, 15 abdominal and 15 CT angiographic patient scans [body mass index (BMI) range: 17 to 37 kg/m^2^] were retrospectively analyzed to determine the CNR at different VMI energies.

**Results:**

Contrast at a given iodine concentration and VMI energy was independent of phantom size, radiation dose, and acquisition voltage (kV). With decreasing VMI energy, the maximum of the NPS curves increased, while their shape remained similar, indicating higher noise but similar noise texture. The CNR increased with lower VMI energy for a given iodine concentration and phantom size, while CNR decreased with increasing phantom size for a given VMI energy and iodine concentration. When the VMI energy was lowered by 5 keV steps in the range of 60 to 40 keV, similar CNR could be maintained when reducing the iodine concentration at each step by 11.7% to 13.7% for abdominal PVP scans and 11.8% to 14.5% for CTAs. CNR analysis of the patient scans confirmed these findings: a 5 keV reduction in VMI energy led to a mean±SD 11.4%±0.4% and 13.7%±1.0% increase in CNR for abdomen PVP and CTA scans, respectively. This can be translated to a corresponding reduction in CM dose when a constant CNR is aimed for. From these results, a simple, robust rule-of-thumb was derived, the 10-to-5 rule: For the evaluated PCD-CT protocols, CNR can be maintained with about 10% less CM dose for each reduction of the VMI energy by 5 keV.

**Conclusions:**

This phantom study, which was complemented with a retrospective proof-of-principle patient study, showed that a simple, easy to implement 10-to-5 rule-of-thumb might be used in daily practice for contrast-enhanced PCD-CT. It allows for individual adaptation of the CM dose to the VMI energy applied.

Approximately 300 million computed tomography (CT) scans are performed worldwide each year—40% of them with administration of iodinated contrast media (CM).^1^ Although possible side effects such as the occurrence of contrast-induced acute kidney injury (CI-AKI) are controversially discussed,^2,3^ it is recognized practice to reduce the administered CM dose as much as clinically feasible.

For CT with energy-integrating detectors (EID-CT), low-kV scanning at 70 to 100 kV has been established as an effective technique for optimizing the overall CM dose: Low tube voltages result in increased iodine contrast and improved iodine contrast-to-noise ratio (CNR), allowing for a reduction in CM dose and/or radiation dose.^4–6^ Previous research^7^ showed that a simple 10-to-10 rule can be used in this respect. In brief, the 10-to-10 rule indicates that the CM dose can be lowered by 10% for each reduction of the tube voltage by 10 kV steps. The lowest feasible kV-value is chosen based on the body habitus of the patient, the planned examination, and the available tube power reserves, for example, by using automated tube voltage selection techniques. The kV-based CM dose reduction is a valuable adjunct to a classic weight-based adjustment of a given CM protocol. The 10-to-10 rule has been shown to be feasible in daily clinical practice,^7,8^ in our institute as well as in others.^9^

As an alternative to low-kV scanning, the reconstruction of virtual monoenergetic images (VMI) at lower energy levels (in keV) has been proposed for dual-energy EID-CT.^10^ Thanks to refined image processing,^11^ VMIs at low keV show similar improvements in the iodine CNR and allow a reduction of the CM dose.^12,13^ The use of VMIs at low keV, however, has not become part of clinical routine, as the acquisition of dual energy data requires special scan modes with limitations on most EID-CT systems, for example, in terms of scanning speed or maximum field-of-view.

Recently introduced first-generation photon-counting detector CT (PCD-CT) routinely acquires spectral data in 2 or more energy bins.^14^ Scanning at a fixed tube voltage of 120 kV or 140 kV and reconstruction of VMIs are recommended for routine applications.^15,16^ The VMI energy is adapted to the clinical application—for example, low energy reconstruction (down to 45 keV) has been proposed for CT angiography (CTA) and 55 to 60 keV for organ studies, respectively. The variation of the keV level in PCD-CT plays a similar role to the variation of the x-ray tube voltage in EID-CT with respect to the contrast enhancement and iodine CNR. Several studies have already demonstrated a possible reduction of the CM dose with PCD-CT when using VMIs at low energy.^17–22^

The increase in CNR with decreasing VMI energy has been demonstrated in patient studies with PCD-CT.^23–25^ Yet, to the best of our knowledge, the underlying relationship between appropriate keV levels and corresponding CM dose has not been systematically investigated. In this context, the question arises whether the 10-to-10-rule for kV-adaptation can be translated to a similar rule for keV-adaptation with PCD-CT, with the aim to further reduce CM dose while maintaining diagnostic image quality.

Therefore, the aim of this phantom study is to transfer the 10-to-10 rule (as established for EID-CT) into a corresponding adaptation in PCD-CT. To this end, iodine CNR in VMIs with different energies (40 to 60 keV in steps of 5 keV) at different iodine concentrations, different phantom sizes, and different radiation doses were systematically investigated, for both an abdominal protocol and a CT angiographic (CTA) protocol. Fifteen abdominal and 15 CT angiographic patient scans were retrospectively analyzed to show a proof of principle.

## MATERIALS AND METHODS

### Phantom

**FIGURE 1:**
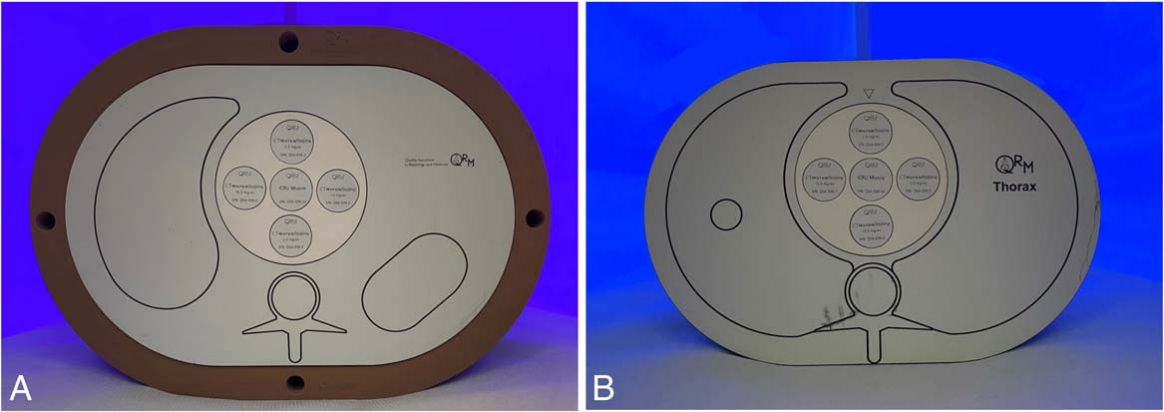
CT phantoms (QRM GmbH, Möhrendorf) with rods in the 5 holes. A, The abdomen phantom with the thin fat ring and rods of configuration 1. B, The chest phantom without ring and rods of configuration 2.

Spectral CT abdominal and chest phantoms (QSA-543/TRX-100; QRM, Möhrendorf, Germany), each with a size of 20×30 cm, were used. To mimic different patient sizes, the phantoms were scanned without and complemented with fat-equivalent rings, resulting in dimensions of 25×35 cm (thin ring) and 30×40 cm (thick ring). Both phantoms had a central 10 cm bore, which was filled with an in-house 3D printed tissue equivalent cylinder (83 HU at 55 keV) with 5 cylindrical holes (2 cm diameter). In these holes, various rods were inserted containing iodine at different concentrations in water-equivalent material (0.5/1.0/2.0/5.0/10.0/15.0 mg I/mL) and a muscle-equivalent material (QRM, Möhrendorf, Germany), as defined by the International Commission on Radiation Units and Measurements ICRU.^26^ As each phantom could hold 5 rods at a time only, all scans were performed in 2 configurations: (1) 0.5/1.0/2.0/10.0 mg I/mL and ICRU muscle tissue, and (2) 1.0/5.0/10.0/15.0 mg I/mL and ICRU muscle tissue (Fig. 1). The phantoms were aligned such that the iso-centrum of the phantom aligned with the iso-centrum of the PCD-CT.

### Computed Tomography Data Acquisition and Image Reconstruction

All scans were performed on a first-generation dual-source PCD-CT (NAEOTOM Alpha, software version VB10; NAEOTOM Alpha, software version VB10; Siemens Healthineers AG, Forchheim, Germany), using 2 standard protocols used in our institution: an abdomen protocol for portal venous phase (PVP) imaging and a high-pitch CTA protocol for visualization of the aorta before a transcatheter aortic valve implantation (TAVI) procedure. The relevant scan and image reconstruction parameters are summarized in Table 1. Scans were performed at the vendor-recommended image quality (IQ) levels of 145 (abdomen PVP protocol) and 64 (high-pitch CTA protocol), at x-ray tube voltages of 120 kV and 140 kV. To account for different preferences of different institutions, we performed scans at 50%, 75%, 100%, 125% and at 150% of the nominal radiation doses, that is, at IQ levels 73, 109, 145, 181, and 218 for the abdominal protocol, and at 32, 48, 64, 79, and 96 for the high-pitch CTA protocol. For each scan, we reconstructed VMIs at different energy levels, focusing on a range around the manufacturer’s preset values (60 keV for abdominal scans, 55 keV for CTAs). Therefore, we reconstructed VMIs from 40 to 60 keV, in steps of 5 keV.

**TABLE 1:** Scan and Image Reconstruction Parameters for the Abdomen PVP and CTA Phantom and Patient Scans

	Abdomen PVP	CTA
kV	120/140	120/140
IQ level	145	64
CAREkeV optimized for	Soft tissue with contrast	Vascular
CAREDose4D	On	On
Pitch	0.8	2.4
Collimation (mm)	144×0.4	144×0.4
Rotation time (s)	0.5	0.25
FOV (mmxmm)	300×300	300×300
Matrix	512×512	512×512
Kernel	Qr40 (patients)/Br40 (phantom)	Qr40
QIR strength	3	3
Spectral recon	Monoenergetic	Monoenergetic
Slice thickness (mm)	2 (patients)/3 (phantom)	1.5
Increment (mm)	1.5 (patients)/2 (phantom)	1.2 (patients)/1.5 (phantom)

The image quality (IQ) level is a vendor-specific parameter for the radiation dose that compensates for different scanner geometries and kV settings. CAREkeV is a vendor-specific task-dependent radiation dose adaptation. CAREDose4D is a vendor-specific anatomic tube current modulation.

FOV indicates field of view; QIR, quantum iterative reconstruction.

### Image Analysis

Images were evaluated with in-house written MATLAB scripts (The MathWorks Inc., Natick, MS, USA). For each image stack belonging to a particular combination of scan and reconstruction parameters, 5 circular regions-of-interest (ROI) were placed in the 5 rods of the phantom. Each ROI was extended along 11 (abdomen) and 16 (chest) image slices to obtain a volume-of-interest (VOI). The mean and SD of the CT values [in Hounsfield units (HU)] were calculated for each VOI. Subsequently, contrast and CNR were determined according to


(Equ. 1) $$\mathrm{contrast}={\mathrm{mean}}_{\mathrm{iodine}}-{\mathrm{mean}}_{\mathrm{background}}$$

(Equ. 2) $$\mathrm{CNR}=\;\frac{{\mathrm{mean}}_{\mathrm{iodine}}-{\mathrm{mean}}_{\mathrm{background}}}{{\mathrm{SD}}_{\mathrm{background}}}$$

For contrast and CNR, the muscle VOI was used as background material.

To not only assess the SD but also the structure of the image noise, the noise power spectrum (NPS) was determined for all abdominal scans using an in-house MATLAB script, see the Appendix, Supplemental Digital Content (http://links.lww.com/RLI/B81). The NPS could not be determined for the CTAs because the chest phantom does not contain sufficiently large homogeneous areas.

The data were transferred to Excel to plot contrast and CNR as a function of the iodine concentration, with each keV level as a separate line. Because both contrast and CNR are expected to depend linearly on the iodine concentration, linear fits to the measured values were performed, and the slope and intercept were calculated. Independent plots were generated for each phantom size, x-ray tube voltage (kV), and radiation dose level. From the graphs of CNR as a function of iodine concentration with keV level as a parameter, we deduced how a reduction in iodine concentration could be compensated by reconstructing VMIs at lower keV to maintain a constant CNR. Thereby, we assume that a reduction in iodine concentration is equivalent to a reduction in CM dose. The iodine concentrations *c*(*x*) required to maintain the constant CNR at each keV-level *x* were calculated from the slopes and intercepts of the linear fits. Then, starting at 60 keV, the percent iodine reduction ∆c(x) per 5 keV reduction in VMI energy at maintained CNR was calculated as follows:


(Equ. 3) $$∆c(x)\;=\frac{c\left(x-5\mathrm{keV}\right)-c(x)}{c(x)}\cdot 100\%$$

where x is 60, 55, 50, or 45 keV.

The percentage iodine reduction ∆c(x) per 5 keV is reported for both abdominal and CTA scans, for a CNR of 5 for abdomen PVP and 15 for CTA. In the literature, CNR values of about 5 have been reported for abdominal PVP examinations^17,27^ and of about 13 for CTA.^28^ The results for 100% radiation dose are shown. To check the general validity of these results independent of the radiation dose, ∆c(x) was calculated for all other relative radiation doses as well, and the maximum deviation from the mean value is reported. Also, the influence of the choice of the CNR value was investigated by repeating the analysis for 100% relative radiation dose and CNR values of 2.5/10/15/20. Again, the maximum deviation from the mean value is reported as an indicator of the validity of the results at different CNRs.

### Proof-of-Principle in Clinical Abdomen Portal Venous Phase Scans and Computed Tomography Angiography Scans

As proof of principle, we retrospectively analyzed CNR in abdominal PVP scans and in CTAs of the aorta of N = 15 patients each. The patients were selected from consecutive examinations so that 5 each could be assigned to 3 different weight categories, with body mass index (BMI) <25, ≥25 and <30, and ≥30 kg/m^2^. The patients were scanned with the protocols described in Table 1 at 120 kV tube voltage. Iodinated CM (Ultravist, 300 mg/ml; Iopromide, Bayer Healthcare, Berlin, Germany) was administered according to a total-body-weight (TBW) adapted protocol with 0.3 gI/kg^17^ and 0.16 gI/kg for abdomen PVP and CTA, respectively. The CM injection duration was standardized at 30 and 14 seconds, respectively, regulated by dedicated CM injection software (P3T; Bayer Healthcare, Berlin, Germany). Consequently, flow rate (in ml/s) was based on patient’s weight and iodine concentration. For abdomen PVP, the scan was initiated ∼70 seconds after injection, where the exact timing was optimized by using bolus tracking. Conversely, for CTA, a 2-second test bolus was used to determine the optimal scan delay, defined as the time to peak enhancement plus 2 seconds.

VMIs were retrospectively reconstructed at energies of 40 to 60 keV in steps of 5 keV using the spectral postprocessing (SPP) images. For each patient, we selected a representative image slice and placed circular ROIs in the right and left liver lobes in the abdomen PVP scans and the ascending and descending thoracic aorta in the CTA scans, and a background ROI in paraspinal muscle tissue. The ROIs were made as large as possible, carefully avoiding blood vessels or pathology. We determined the mean CT-number (in HU) and the SD in each ROI for each keV-level using commercial image processing software (Syngo.via, version VB80, Siemens Healthineers AG, Forchheim, Germany). After averaging the mean CT-numbers of both ROIs in the liver (abdomen) or aorta (CTA), respectively, CNR was calculated according to Equ. 2, using liver/aorta as “iodine” and muscle as “background.” Finally, we calculated the percentage CNR differences ∆CNR(x) between subsequent keV-levels *x* and *x*- 5 keV: 45 to 40 keV/50 to 45 keV/55 to 50 keV/60 to 55 keV according to Equ.3, but with CNR(*x*) instead of *c*(*x*).

### Statistical Analysis

Statistical analyses were performed using Excel. Continuous parametric data such as SD, contrast, or CNR are given as mean ± SD, or as mean and maximum deviation from the mean value in percentage.

### Ethics

The proof-of-principle study received a waiver of written informed consent from the local ethical committee and Institutional Review Board (METC 2024-0471), due to its retrospective nature.

## RESULTS

### Radiation Dose

The weighted computerized CT dose index (CTDI_w,vol;_ [the CTDI_w,vol_ in mGy is a defined measure according to (IEC 60601-2-44) to account for the table feed (pitch) in spiral/helical scans] values for the abdomen PVP protocol and the TAVI high-pitch CTA protocol at the different IQ levels are summarized in Table 2. CTDI_w,vol_ depends on both, the tube voltage and the phantom size. For each IQ level, CTDI_w,vol_ is slightly larger at 140 kV than at 120 kV for the phantoms without ring and with thin ring, but slightly smaller for the phantom with thick ring. The CTDI_w,vol_ values at other IQ levels scale with the relative dose level (50 to 150%), the maximum deviation from the expected value at each dose level is 2.8%.

**TABLE 2:** Radiation Dose (CTDI_w,vol_ in mGy) of the Scans With the Abdomen PVP Protocol and the High-pitch CTA Protocol, at the Standard IQ level (145 for Abdomen and 64 for CTA), for Different Phantom Sizes and Tube Voltages

	No Ring		Thin Ring		Thick Ring	
Tube voltage (kV)	120	140	120	140	120	140
Abdomen PVP	4.56	4.78	7.36	7.33	11.2	10.8
CTA	0.92	1.01	1.42	1.45	2.42	2.37

### Phantom Scans—Abdomen Portal Venous Phase Protocol

**FIGURE 2:**
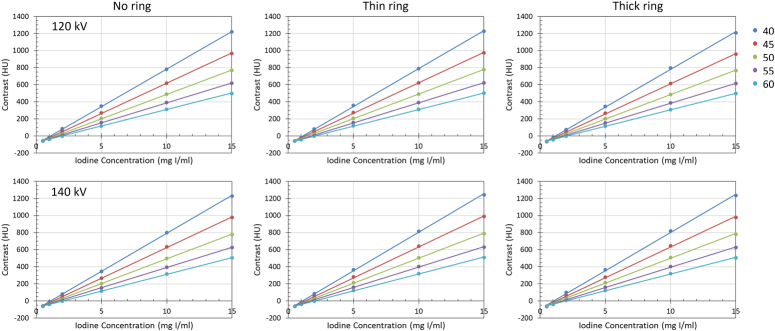
Contrast in Hounsfield Units (HU) as a function of the iodine concentration for the abdomen PVP protocol at 120 kV (top row) and at 140 kV (bottom row), both at 100% radiation dose, with the VMI-energy in keV as a parameter. Left column: no ring; middle column: thin ring; right column: thick ring. The dots are measured values, the solid lines are linear fits to the measurements and correspond to the keV levels from 40 keV to 60 keV in steps of 5 keV.

Figure 2 shows the measured contrast according to Equ. 1 as a function of the iodine concentration for the abdomen PVP protocol. For each keV, the contrast increases linearly with the iodine concentration. The contrast at a given iodine concentration and VMI energy is independent of the phantom size, relative radiation dose, and acquisition voltage (kV). For each iodine concentration, the contrast increases with decreasing VMI energy. The negative contrast at low VMI energies is a consequence of using muscle tissue as the background material in Equ.1, whereas the iodine is contained in water-equivalent tissue. However, this only produces a constant offset, which is irrelevant for comparisons of contrast and CNR at different parameters.

The measured NPS curves as a function of spatial frequency are very similar for all VMI energies (Appendix, Supplemental Digital Content, http://links.lww.com/RLI/B81). Their maximum increases with decreasing keV, indicating higher image noise at lower VMI energy. The spatial frequency of the maximum does not change with decreasing VMI energy for the phantoms without ring and with thin fat ring. For the phantom with thick fat ring, the maximum shifts to lower spatial frequencies, from 60 to 40 keV by about 0.03 lp/mm. This indicates a similar noise texture at all VMI energies for slim and normal-sized patients, and a slightly coarser noise texture at lower VMI energies for patients with higher BMI.

**FIGURE 3:**
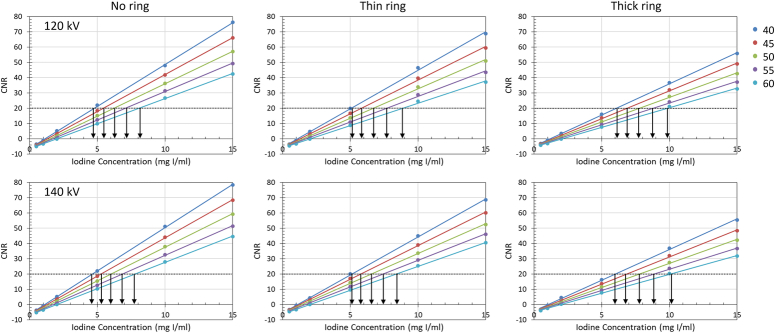
Contrast-to-noise ratio (CNR) as a function of the iodine concentration for the abdomen PVP protocol at 100% radiation dose at 120 kV (top row) and at 140 kV (bottom row), with the VMI-energy in keV as a parameter. Left column: no ring; middle column: thin ring; right column: thick ring. The dots are measured values, each solid line is a linear fit to the measurements at the respective keV level. Merely for better visualization, a CNR of 20 instead of 5 was chosen here, which is marked by a horizontal line. The arrows show the iodine concentrations required to obtain this CNR at the different keVs (intersection points of the lines for the different keVs with the horizontal line).

Figure 3 shows the measured CNR according to Equ. 2 as a function of the iodine concentration for the abdomen PVP protocol. For each phantom size and each keV, CNR increases linearly with the iodine concentration. For each iodine concentration and each phantom size, the lower the VMI energy, the higher the CNR. This shows that the contrast increases more than the image noise at lower VMI energy. Overall, CNR decreases with increasing phantom size. A comparison of CNR values for 120 kV and 140 kV shows that CNR for a given phantom size, iodine concentration, and VMI energy depends only marginally on the x-ray tube voltage. In Figure 3, arrows illustrate how the iodine concentration can be reduced when the VMI energy is reduced by 5 keV while maintaining a constant CNR.

The iodine concentrations *c*(*x*) to maintain a constant CNR of 5 at 100% radiation dose with the abdomen PVP protocol at VMI energies *x* decreasing from 60 to 40 keV in steps of 5 keV are listed in Table 3, for all phantom sizes at both 120 kV and 140 kV. Table 3 also shows the percentage iodine reductions ∆c(x) per 5 keV reduction in VMI energy according to Eq. 3.

**TABLE 3:**
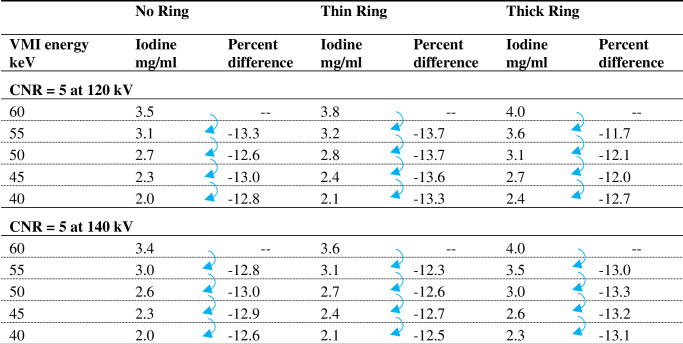
Iodine Concentrations to Maintain a Constant CNR of 5 With the Abdomen PVP Protocol at 100% Radiation Dose When the VMI Energy Decreases From 60 keV to 40 keV in Steps of 5 keV, for the Phantoms Without Ring, With Thin and With Thick Ring

Top: 120 kV, bottom: 140 kV. The percentage difference refers to the transition from the upper to the lower keV level, indicated by a blue arrow. See also Equ. 3.

For the same VMI energy, a higher iodine concentration is needed for thicker phantoms to maintain the desired CNR. For each phantom size, the required iodine concentration decreases with decreasing keV of the VMIs. The percentages ∆c(x) do not depend on the relative radiation dose level and the CNR value: for the relative radiation doses from 50% to 150%, the maximum deviation of ∆c(x) from the mean value is -1.4% and +0.3%. Variation of the set CNR value from 2.5 to 20 results in a maximum deviation from the mean value of -2.3% and + 1.7%.

### Phantom Scans—Computed Tomography Angiography Protocol

**FIGURE 4:**
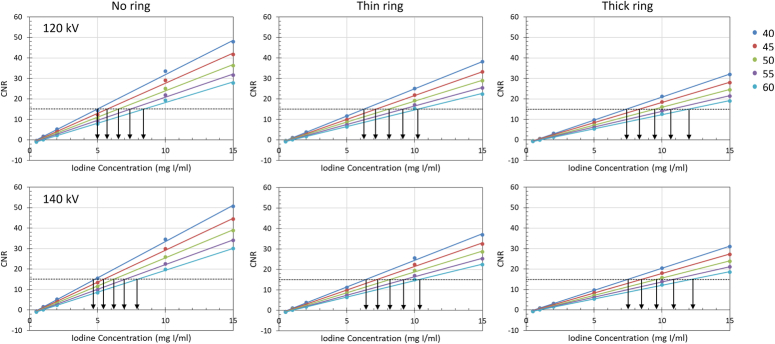
Contrast-to-noise ratio CNR as a function of the iodine concentration for the high-pitch CTA protocol at 100% radiation dose at 120 kV (top row) and at 140 kV (bottom row), with the VMI-energy in keV as a parameter. Left column: no ring; middle column: thin ring; right column: thick ring. The dots are measured, each solid line is a linear fit to the measurements at the respective keV level. The CNR of 15 is marked by a horizontal line. The arrows show the iodine concentrations required to obtain this CNR at the different keVs (intersection points of the lines for the different keVs with the horizontal line).

As with the abdomen PVP protocol, contrast increases linearly with the iodine concentration and is independent of the phantom size, relative radiation dose, and acquisition voltage (kV) at a given iodine concentration and VMI energy. Figure 4 shows the measured CNR according to Equ. 2 as a function of the iodine concentration for the high-pitch CTA protocol.

CNR increases linearly with the iodine concentration, decreases with increasing phantom size, and is independent of the X-ray tube voltage for any phantom size. The lower the VMI energy, the higher the CNR for a given iodine concentration and phantom size.

The iodine concentrations *c*(*x*) to maintain a constant CNR of 15 with the CTA protocol at 100% radiation dose at VMI energies *x* decreasing from 60 keV to 40 keV in steps of 5 keV are listed in Table 4, for all phantom sizes at both 120 kV and 140 kV. Table 4 also shows the percentage iodine reductions ∆c(x) per 5 keV reduction in VMI energy according to Eq. 3.

**TABLE 4:**
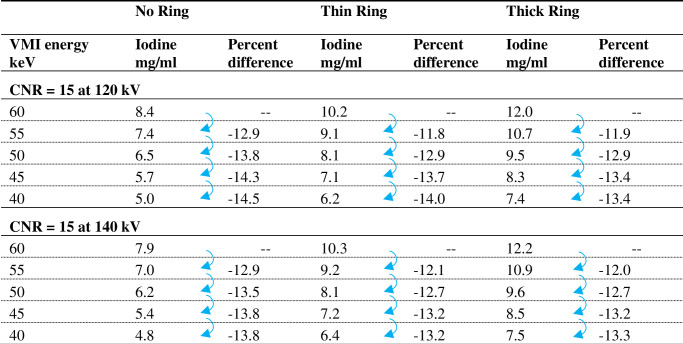
Iodine Concentrations to Maintain a Constant CNR of 15 With the CTA Protocol at 100% Radiation Dose When the VMI Energy Decreases From 60 keV to 40 keV in Steps of 5 keV, for the Phantoms Without Ring, With Thin Ring and With Thick Ring

Top: 120 kV, bottom: 140 kV. The percentage difference refers to the transition from the upper to the lower keV level, indicated by a blue arrow. See also Eq. 3.

For the same VMI energy, a higher iodine concentration is needed for thicker phantoms to maintain the desired CNR. For each phantom size, the required iodine concentration decreases with decreasing keV of the VMIs. The percentages ∆c(x) do not depend on the relative radiation dose level and CNR, with a maximum deviation from the mean of -1.6% to +0.1% for variation of the radiation dose from 50% to 150%, and -1.4% to +2.5% for variation of CNR from 2.5 to 20.

### Practical Rule-of-Thumb

For all phantom sizes and X-ray tube voltages (120 kV or 140 kV), a simple practical rule-of-thumb can be derived from Tables 3 and 4. It applies to a VMI energy range from 40 keV to 60 keV:

→ “10-to-5 rule”: the same CNR is achieved with about 10% less iodine when the VMI energy is reduced by 5 keV.

This rule-of-thumb is conservative, as the percentages of possible iodine reduction are higher than 10% in Tables 3 and 4.

### Proof-of-Principle in Patients

**FIGURE 5:**
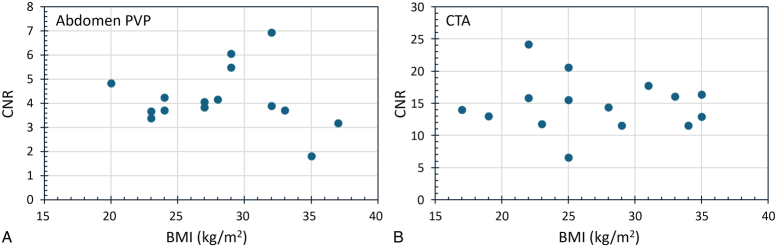
Contrast-to-noise ratio CNR as a function of the body mass index BMI for 15 patients scanned with the standard abdomen PVP protocol (A) and the high-pitch CTA protocol (B), for VMIs at 55 keV.

Figure 5 shows CNRs of the 15 abdominal and the 15 CTA patient scans calculated according to Equ. 4 for VMIs at 55 keV as a function of the BMI. The mean±SD CNRs of 4.2±1.2 (range: 1.9 to 6.9) for abdominal scans and of 15±4 (range: 7 to 24) for CTAs agree well with the literature.^17,24,25^ Although variation between patients was observed, no functional dependence of CNR on BMI is apparent.

**FIGURE 6:**
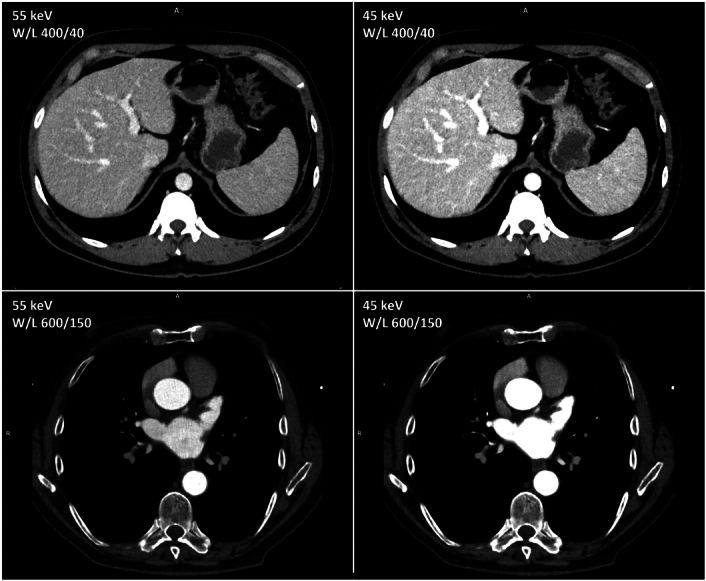
Example images of an abdomen PVP (upper row) and CTA (bottom row) scan reconstructed at 55 keV (left) and 45 keV (right). The BMI of the abdomen PVP patient was 27.8 kg/m^2^, and of the CTA patient 25 kg/m^2^. CNR increased from 4.18 to 5.04 (+20.6%) for the abdomen PVP, and from 20.6 to 26.1 (+27%) for the CTA, for the 10 keV decrease in VMI energy.

Figure 6 shows CT images of an abdominal scan and a CTA from the 2 patient groups reconstructed at 2 different keV levels (55 keV and 45 keV). It illustrates that at lower keV the contrast increases more than the image noise, resulting in increased CNR.

**FIGURE 7:**
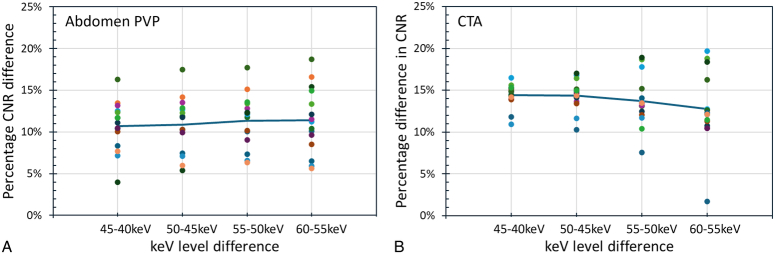
Absolute percentage difference in contrast-to-noise ratio CNR for 5 keV intervals in VMI energy. The dots represent n = 15 patients scanned with the abdominal PVP protocol (A) and the CTA protocol (B), the solid line is the average increase per 5 keV difference interval.

Figure 7 shows for all 15 patients the absolute percentage CNR differences between the VMIs at successive keVs, namely 45 to 40 keV, 50 to 45 keV, 55 to 50 keV, and 60 to 55 keV. The percentage differences vary between patients, but per patient, they are relatively constant among the different keV level differences. For both the abdomen PVP scans and the CTA scans, the percentage CNR increase does not depend on the keV-level or CNR value or BMI. For the abdomen PVP scans, the mean percentage CNR increase is 11.4% ± 0.4% (mean ± SD) per VMI energy decrease of 5 keV, while for the CTAs this percentage is 13.7 ± 1.0%. A CNR increase of 11.4%, respectively 13.7% implies that the amount of contrast agent could be reduced by 11.4%, respectively 13.7% without a reduction in CNR. This is in good agreement with the phantom scans and supports the practical “10-to-5 rule-of-thumb".

## DISCUSSION

This study shows that in both abdominal scans and CTAs performed on a PCD-CT, iodine load can be reduced while maintaining a constant CNR for VMIs reconstructed at a lower energy. Accordingly, a simple rule was derived: reducing the VMI energy by 5 keV allows a 10% reduction in iodine load with preserved CNR. We hypothesize that this finding can be transferred to a corresponding reduction of the CM dose in patient scans. The “10-to-5 rule” applies to the energy range of 40 to 60 keV. The rule-of-thumb is conservative, as the percentage differences in Tables 3 and 4 are higher than 10%, indicating that potentially a slightly larger iodine reduction would be possible.

The phantom results are supported by retrospective proof-of-principle CNR analysis in 30 patient scans. Here, the CM dose is unchanged per patient, but it was shown that a 5 keV reduction in VMI energy leads to a mean 11.4% and 13.7% increase in CNR for abdomen PVP and CTA scans, respectively, with no systematic dependence on the BMI of the patients. This may be a consequence of using total body weight-adapted CM injection protocols. The CNR increase can be translated to a corresponding reduction in CM dose when a constant CNR is aimed for. The somewhat lower average CNR of 4.2 compared with literature values of 5 for the abdomen PVP protocol can be explained by the 2 mm slice thickness used for CNR analysis instead of the clinically used 3 mm. Because the CNR analysis was done retrospectively, the SPP images had to be used with a maximum slice thickness of 2 mm.

As a practical application of the “10-to-5 rule,” CTA scans can potentially be performed with 20% lower CM dose when reconstructing VMIs at 45 keV instead of the vendor-proposed 55 keV (10 keV reduction). However, future studies need to be performed to investigate this further.

In conventional EID-CTs, low-kV scanning is an established method to reduce the CM dose in CTAs, but also in abdominal scans. For the clinically approved PCD-CT, scanning at a fixed x-ray tube voltage of 120 kV or 140 kV and reconstruction of VMIs at typical 55 to 60 keV is recommended instead for routine contrast-enhanced protocols. In EID-CT, the so-called 10-to-10 rule^7^ is successfully used,^8^ which suggests a reduction of the CM dose by 10% when reducing the tube voltage by 10 kV. This rule is supplemented here by a corresponding rule-of-thumb for low-keV imaging with PCD-CT, the “10-to-5 rule.” With low-kV EID-CT, the tube voltage is set before the scan, and the CM dose is adjusted accordingly. However, patients with a higher BMI may not benefit from low-kV scanning due to limited x-ray tube power. In case of suboptimal results (eg, insufficient contrast or high noise), retrospective adjustment is not possible. With low-keV PCD-CT, the VMI energy is also set before the scan, and the CM dose is adjusted correspondingly. Using lower keV levels is not limited to patients with lower BMI for the evaluated protocols—our phantom results were independent of the phantom size, and in the proof-of-principle patient data, the BMI did not systematically influence either CNR or CNR increase with decreasing VMI energy. Furthermore, although it is not possible to change the CM dose retrospectively if the results are suboptimal, it is possible to modify the keV level to improve the contrast or reduce the image noise. However, these findings will need to be confirmed in larger clinical studies.

In a recent study on a PCD-CT,^20^ the authors used a dynamic circulation phantom to investigate the possible reduction in CM dose for coronary CTA when VMIs were reconstructed at lower keV levels instead of a reference energy of 70 keV. VMI reconstructions at 40 to 55 keV at 75% and 40 keV at 50% CM dose provided similar or higher attenuation, vessel sharpness, and CNR compared with the reference protocol. A 50% reduction in CM dose at 40 keV compared with 70 keV is in reasonable agreement with our findings, when they are extrapolated from 60 keV to 70 keV. However, the authors did not establish a systematic rule for CM dose reduction as a function of VMI energy, as was done in our study.

Other studies have demonstrated potential CM dose reduction on PCD-CT compared with EID-CT. In a recent study using a similar phantom configuration,^29^ the CNR of iodine was measured as a function of VMI energy, but the study had a different objective. The authors compared the CNR increase of PCD-CT to EID-CT for the same iodine load at the same VMI energy. They found a mean increase ranging between 48.9 ± 1.6% at 40 keV and 24.4 ± 1.1% at 70 keV and translated this increase into a corresponding potential reduction of the CM dose. Various clinical studies have investigated the potential reduction in the CM dose with PCD-CT, either in comparison to the same or a similar patient group on EID-CT^17,19,30,31^ or by comparing different contrast protocols on PCD-CT.^18,32^

Our study has some limitations. To adjust the CM dose to the VMI energy, we have chosen the preservation of CNR as the only criterion. Based on previous experience, this is adequate for CTAs. For abdominal PVP scans, however, it should be noted that the lower the keV level, the higher the contrast, but also the image noise. This can be challenging when assessing parenchymal organs and requires further patient studies to confirm the practical applicability of the “10-to-5 rule” in this case. However, similar limitations also apply to low kV scanning following the 10-to-10 rule in EID-CT. Furthermore, the analysis of the NPS indicated a similar noise texture at all VMI energies for small and normal-sized patients, which eases image interpretation at lower keV. It remains to be investigated whether the somewhat coarser noise texture for patients with higher BMI can affect the acceptance of low keV images in abdominal PVP CT. However, the reconstruction of VMIs with lower energy than the 60 keV suggested by the manufacturer for abdominal imaging, has been proposed by other authors as well, both for adult^15^ and pediatric patients.^33^ In this case, the “10-to-5 rule” provides simple instructions on how to further optimize the CM dose compared with the CM protocol used at 60 keV. Apart from CNR and NPS, we did not examine other image quality parameters, such as different beamhardening artifacts at different VMI energies, which could influence the performance of the readers. This phantom study is based on 2 clinical acquisition protocols, only—a routine abdominal PVP protocol and a high-pitch CTA protocol. The applicability and robustness of the “10-to-5 rule” to other protocols and the 24/7 setting as well should, therefore, be investigated in further studies. Lastly, we have evaluated one dedicated commercially available PCD-CT of one manufacturer—whether the “10-to-5 rule” will also be applicable to PCD-CT systems of other manufacturers remains to be seen.

In conclusion, this phantom study, which was complemented with a proof-of-principle retrospective patient study, showed that a simple “10-to-5 rule-of-thumb” could be derived to adapt the CM dose to the VMI energy for contrast-enhanced PCD-CT: lowering the VMI energy by 5 keV enables 10% CM dose reduction. This rule applies to the clinically relevant energy range of 40 to 60 kV for abdominal PVP and CTA scans. It allows for optimization of CT and CM protocols, which are intertwined.

## Supplementary Material
