# Supplementary material for: A Practical Rule-of-Thumb to Adapt Contrast Media Dose in Photon-counting Detector CT: The 10-to-5 Rule
Source: Invest Radiol. 2025 Nov 26;61(8):527–36. doi: 10.1097/RLI.0000000000001253 (PMC13317918; doi:10.1097/RLI.0000000000001253)

**Appendix**

The noise power spectra (NPS) were measured in subtraction images of the abdomen phantom at adjacent z-positions using the largest possible rectangular ROI in a homogeneous region, see Figure A1. The noise power spectra of 11 consecutive difference images were averaged to obtain the final NPS curves.

Figure A2 shows the calculated noise power spectra (NPS) as a function of spatial frequency (in lp/mm) for the abdomen PVP protocol at 50% radiation dose, for the abdomen phantom without ring, with thin and thick fat ring, at both 120 kV and 140 kV. The solid lines correspond to VMI energies from 40 keV to 60 keV in steps of 5 keV. 50% radiation dose was chosen because at higher image noise the NPS calculation is less affected by unwanted side effects.

The NPS curves are similar for all keV levels and phantom sizes. For a given phantom size, both the maximum and the area under the NPS curve increase with decreasing VMI energy – this indicates higher image noise at lower keV. As the phantom size increases, the NPS curves become higher - the automatic dose modulation adjusts the radiation dose to the phantom size, but not with the aim of constant noise. Instead, higher image noise is tolerated with larger phantoms. The spatial frequency of the maximum of the NPS curves doesn’t change with decreasing VMI energy for the phantoms without ring and with thin fat ring. For the phantom with thick fat ring, a slight shift by about 0.03 lp/mm towards lower frequencies can be observed for VMI energies from 60 keV to 40 keV. This indicates a similar noise texture at all VMI energies for slim and normal sized patients, and a slightly coarser noise texture at lower VMI energies for patients with higher BMI.

**Figure A1:** Noise power spectra NPS as a function of the spatial frequency in lp/mm for the abdomen PVP protocol at 50% radiation dose at 120 kV (top row) and at 140 kV (bottom row), convolution kernel Br40, QIR 3. Left: no ring; middle: thin ring; right: thick ring.


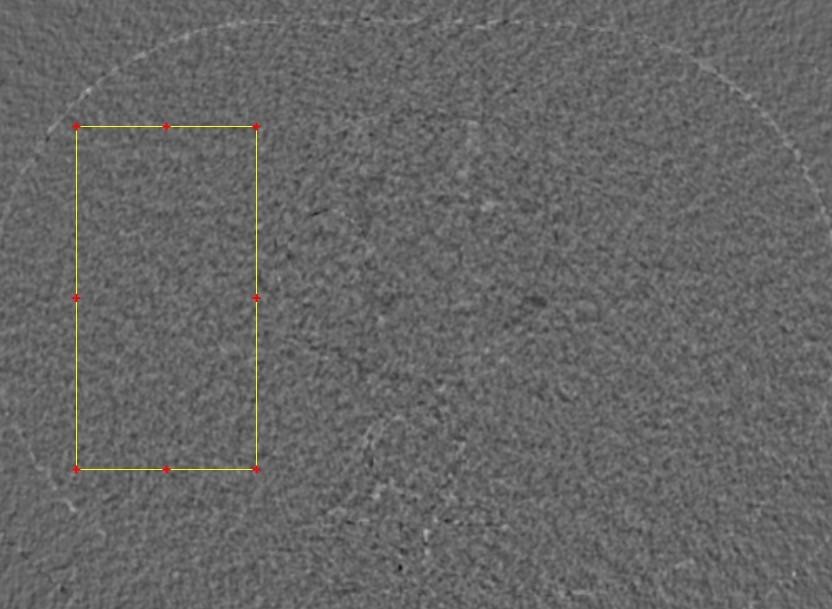


**Figure A2:** Noise power spectra NPS as a function of the spatial frequency in lp/mm for the abdomen PVP protocol at 50% radiation dose at 120 kV (top row) and at 140 kV (bottom row), convolution kernel Br40, QIR 3. Left: no ring; middle: thin ring; right: thick ring.


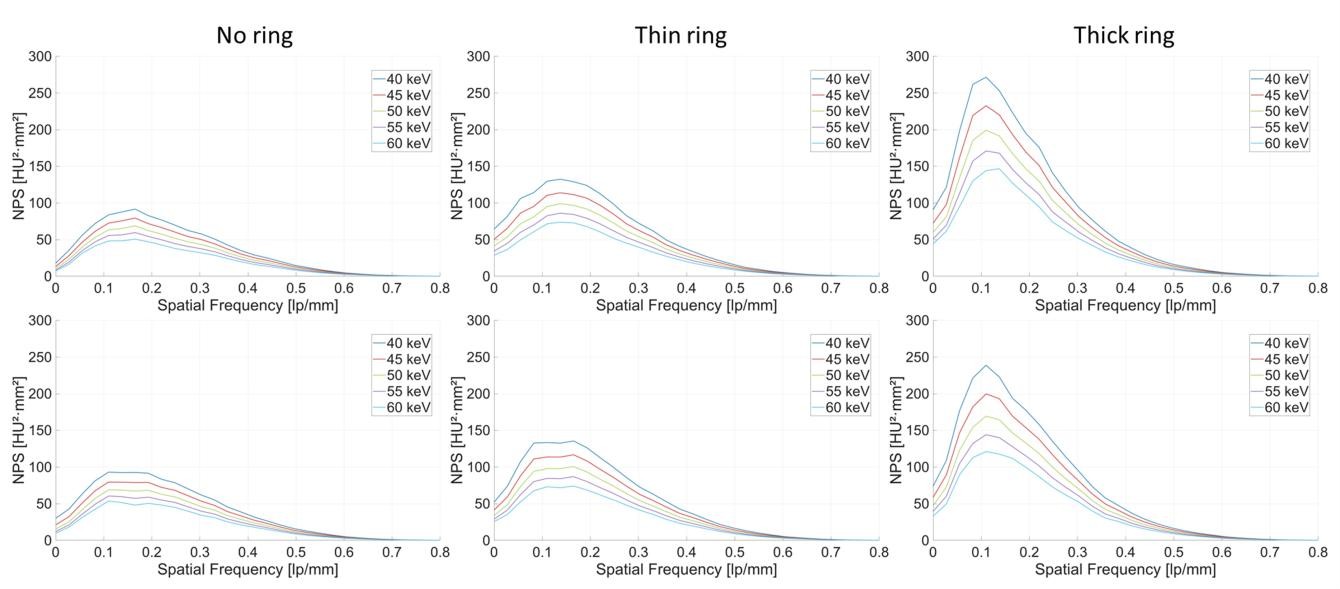

Supplement: Supplementary file 1 [file rli-61-527-s001.docx]
